# Change in the Air: How Shifting Federal Guidance Related to DEI Influences Teachers’ Use of Culturally Responsive Practices

**DOI:** 10.3390/bs16030390

**Published:** 2026-03-09

**Authors:** Kate M. Morman, Laura M. Brady, Cong Wang, Stephanie A. Fryberg

**Affiliations:** 1 Department of Psychology, Northwestern University, Evanston, IL 60208, USA; cong.wang@northwestern.edu (C.W.); fryberg@northwestern.edu (S.A.F.); 2 Education Systems and Policy Program Area, American Institutes for Research, Chicago, IL 60606, USA; lbrady@air.org

**Keywords:** culturally responsive practices, diversity ideologies, moral beliefs in education, equity-focused teaching in political contexts

## Abstract

Culturally responsive practices (CRPs) (i.e., practices that affirm students’ cultural backgrounds) can reduce educational inequities, but these practices have yet to become normative within U.S. education. A national study of K-12 teachers conducted in late 2023 found that teachers’ use of CRPs depended not only on their individual moral frameworks regarding diversity (i.e., endorsement of multiculturalism and colorblindness) but also on their communities’ and administrators’ support for efforts to increase equitable educational outcomes among students (i.e., equity work). Since 2023, federal guidance has shifted, and educational equity work is now discouraged. We conducted a second national survey of K-12 teachers (*N* = 980) in early 2025 to examine whether contextual influences on teachers’ decisions regarding CRP use have also shifted in light of changes to federal guidance. While the 2023 study found that teachers with weaker multiculturalism beliefs were more likely to use CRPs when their administrators supported equity work, findings from the 2025 study revealed that administrator support only predicted greater CRP use when these teachers worked in politically liberal (but not conservative) communities. The shift suggests that recent federal policy changes have weakened the influence of district and school leadership on teachers’ decisions to use CRPs, particularly among teachers who are not individually inclined to use these practices. This study offers timely insights into teachers’ use of CRPs after new federal guidance on educational equity efforts and reaffirms that teachers’ practices are not solely shaped by their personal beliefs, but are instead responsive to the broader contexts in which they work.

## 1. Introduction

Over the past 30 years, prioritizing educational diversity, equity, and inclusion (DEI) efforts has been an increasing imperative as educators, researchers, and policy makers have adjusted to an increasingly diverse student body and sought to mitigate racial and socioeconomic achievement gaps (Brady et al., 2024; Improving America’s School Act, U.S. Congress, 1994; Sleeter, 1992a, 1992b). During this time, educational theory and research have emphasized the importance of recognizing students’ cultural backgrounds as assets, rather than deficits, in learning (Gay, 1994; Ladson-Billings, 1995). Many states have integrated this imperative into their professional standards by outlining expectations for teachers to engage students using culturally responsive practices (CRPs; Muñiz, 2019), which refer to instructional practices that affirm and leverage students’ cultural knowledge and experiences to deepen engagement and promote equitable educational outcomes (Gay, 2015; Ladson-Billings, 2014; Paris, 2012; Paris & Alim, 2014). Despite their potential to improve educational quality, CRPs have become controversial as conversations about DEI efforts in education have become increasingly politicized in recent years (LoBue & Douglass, 2023; McGowan et al., 2025). Conversations about the role of DEI in education have brought immense scrutiny to what teachers convey to students and how they structure student learning experiences (Peetz & Najarro, 2024; Pollock et al., 2023; Will & Najarro, 2022). In many communities, teachers are forced to make sense of competing perspectives about how they should conduct their work and which practices and content they should use in their classrooms (Bylica et al., 2025).

Contrary to concerns that teachers’ practices are politically motivated or selected to indoctrinate students with “divisive ideologies” (Murawski, 2021), research illustrates that teachers’ decisions to use CRPs depend upon not only their own beliefs about the importance and efficacy of these practices, but also on the messages of support or opposition for equity work that they receive from their school administrators and local communities (Morman et al., 2025). To unpack the interplay between individual and contextually derived moral frameworks, we begin with individual moral frameworks before detailing contextually derived moral frameworks. For example, at the individual level, teachers’ diversity beliefs (i.e., their widely shared beliefs about the right way to recognize and respond to cultural diversity) are most frequently studied in terms of multiculturalism and colorblindness. Teachers who endorse multiculturalism and thus believe that it is moral or right to acknowledge diversity and cultural differences (Plaut, 2002) are more likely to embrace CRPs as an effective strategy for engaging students (Aragón et al., 2017). On the other hand, teachers who endorse colorblindness and thus believe that it is moral or right to downplay diversity and cultural differences (Neville et al., 2013; Plaut, 2002) are more likely to favor instructional practices that leverage a uniform (i.e., not culturally tailored) approach to engaging students (Castro Atwater, 2008, Turetsky et al., 2021).

Morman et al. (2025) illustrated that teachers often override their individual diversity beliefs when deciding whether to implement CRPs, looking instead to their communities and administrators for guidance. When teaching in communities that were not supportive of educational DEI efforts, both teachers who strongly endorsed multiculturalism (i.e., those typically inclined to use CRPs, referred to as pro-multiculturalism teachers) and those with weaker endorsement (i.e., those typically less inclined to use CRPs, referred to as lean-multiculturalism teachers) were more likely to use CRPs when their administrators supported efforts to advance educational equity (i.e., equity work). When local communities were supportive of DEI efforts, administrators’ support for equity work continued to influence practice decisions among teachers with weaker endorsement of multiculturalism (i.e., lean multiculturalism), who were more likely to use CRPs when their administrators supported equity work. These findings suggest that while teachers’ individual beliefs, administrators’ beliefs, and community-level beliefs all play a role in shaping educators’ use of CRPs, the information teachers receive from administrators plays a particularly important role, especially for teachers whose personal beliefs or community contexts would otherwise lead them to eschew CRPs.

This research paints a nuanced portrait of the individual and contextual factors that influence teachers’ decisions regarding CRPs, but the larger political context has changed in qualitatively meaningful ways since the research was conducted. At the start of 2025, the White House released an executive order (EO) indicating that DEI-related initiatives within the federal government were to be suspended (Executive Order 14151, The White House, 2025). This EO was followed by additional actions within the U.S. Department of Education that had implications for teachers, including the launch of the “End DEI Portal,” a reporting system for “parents, students, teachers, and the broader community to submit reports of discrimination based on race or sex in publicly funded K-12 schools,” (U.S. Department of Education, 2025a) and a memorandum requiring school districts to certify compliance with the cessation of DEI-related activities (U.S. Department of Education, 2025b). These rapid changes in federal guidance regarding educational DEI efforts have generated widespread confusion and heightened concerns among teachers, many of whom are uncertain about which instructional practices remain permissible (Sobre & Seals, 2025). Moreover, in some places, new federal guidance seems to contradict state-level education standards that mandate teaching from multiple perspectives and attending to students’ cultural backgrounds (e.g., California’s ethnic studies standards and Massachusetts’s history standards; Furrey, 2024).

Given these substantive changes in the political landscape, how teachers may weigh new federal DEI guidance alongside their individual and contextually derived moral frameworks when deciding whether and how to use CRPs is unclear. Emerging research suggests that some teachers exercise greater caution about expressing pro-DEI beliefs or implementing CRPs, particularly those who teach in districts that have restricted DEI efforts (Borsheim-Black, 2024). In contrast, teachers in contexts with community-level support for DEI may perceive that the right path forward is to disregard new federal guidance and implement practices that align with their communities’ beliefs. Based on prior results, we anticipate that contextual support in the form of district and school administrator supports will continue to be influential within communities that are opposed to DEI (Hambacher et al., 2024) and that this may be particularly true for teachers with strongly codified multiculturalism beliefs who are more inclined to use CRPs (Hachfeld et al., 2015). Indeed, recent qualitative research suggests that teachers in states with anti-DEI legislation are less constrained in their practice decisions in the presence of either community or administrator support for equity work (Kelly et al., 2024; Stevens et al., 2025).

Furthermore, we also anticipate that when communities are largely supportive or permissive of DEI efforts, the influence of administrator support may continue to be organized by teachers’ individual moral frameworks. Previously, teachers with stronger endorsement of multiculturalism (i.e., pro-multiculturalism teachers) reported relatively frequent use of CRPs, regardless of their administrator support for equity when they taught in local communities that were supportive of DEI efforts (Morman et al., 2025). Consistent with these prior results, we theorize that when the community political climate is supportive of DEI, these teachers may feel free to implement practices that are aligned with their individual moral frameworks, even in the absence of administrator support. For teachers with less codified beliefs (i.e., lean-multiculturalism teachers), we expect that even though their community may be supportive or permissive of DEI efforts, administrator support will continue to play an important influence in their use of CRPs (Morman et al., 2025). Prior evidence indicates that administrator support for equity is cued through normative processes and everyday interactions (Blaushild, 2023; Peurach et al., 2019). To teachers with less codified beliefs, a lack of administrator support for equity may cue inaction as the normative response (Cobb, 2017), while strong administrator support for equity may cue an expectation that teachers use inclusive practices (Hagenaars et al., 2023).

Further examination is required to better understand whether and how new federal guidance may have shifted the balance of individual and contextual influences on teachers’ decision making in their use of CRPs. The current study was designed to explore this question by comparing data collected through a national survey of K-12 teachers in 2025 to data collected in the previously reported national survey of K-12 teachers conducted in 2023 (Morman et al., 2025). We anticipate, in line with emerging research, that when deciding whether to implement CRPs, teachers with less codified beliefs in multiculturalism who teach in communities that are less supportive of DEI efforts may pay greater heed to new federal guidance compared to the messages they receive from their administrators regarding equity work. In contrast, in line with our prior results, we anticipate that teachers who strongly endorse multiculturalism will continue to use CRPs when they experience at least one contextual indicator of support for equity work—either from their administrators or from their communities.

## 2. Materials and Methods

**Figure 1:**
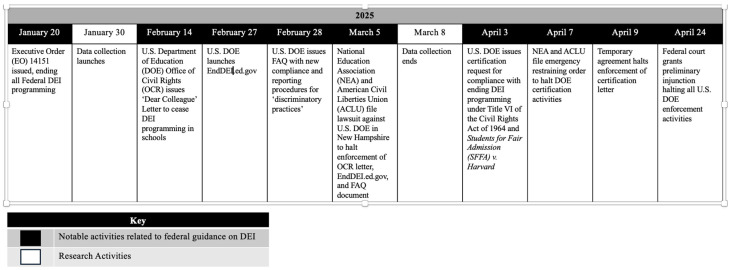
Timeline of notable activities related to federal guidance on DEI and research activities in 2025.

To examine whether and how changes to federal guidance regarding DEI in education shifted the influence of individual and contextual factors on teachers’ decisions to use CRPs, we leveraged two national surveys of using different samples of K-12 educators: the first survey was fielded in 2023, prior to changes in federal guidance regarding DEI, and the second was fielded between 30 January and 8 March 2025. Notably, the 2025 survey was launched 10 days after EO 14151, which outlined the federal government’s new stance on DEI, and data collection continued through several notable changes related to DEI within the Department of Education (see Figure 1 for a timeline of notable events related to changing federal guidance and research activities). The 2023 and 2025 surveys included either identical or comparable measures of teachers’ diversity beliefs (i.e., endorsement of multiculturalism and colorblindness), use of CRPs, perceived administrator support for educational equity work, and community political climate, allowing for an examination of whether and how the individual and contextual influences on teachers’ use of CRPs changed following shifts in federal guidelines. While standalone analyses of the 2023 survey are reported in a prior publication (Morman et al., 2025), the current paper integrates data from the same 2023 survey with data from the 2025 survey to conduct new analyses focused on exploring shifts in individual and contextual influences on teachers’ practices.

### 2.1. Participants

Participants in the 2025 survey included 980 K-12 teachers recruited through Qualtrics Research Panels. The sample included teachers from all U.S. states and the District of Columbia, with the exception of Alaska. Most teachers self-identified as female (78.4%) and White (78.7%). The majority (59.6%) held an advanced degree (i.e., master’s degree) and had an average of 18 years of teaching experience, excluding student teaching. Half of participants (49.5%) taught in suburban schools, 27.1% taught in urban schools, and 22.1% taught in rural schools. On all demographic measures, the 2023 sample (*N* = 1015)^1^, which was majority female (79.3%) and White (77.1%) with 15 years of teaching experience, was comparable to the 2025 sample, apart from the 2023 sample being more likely to have a bachelor’s degree (41.0%) rather than an advanced degree. Half of participants (48.3%) in the 2023 sample taught in suburban schools, 30.0% in urban schools, and 20.5% in rural schools.

### 2.2. Measures

#### 2.2.1. Diversity Beliefs and CRPs

The 2025 survey used the same measures as the 2023 survey to assess diversity beliefs (i.e., endorsement of colorblindness and multiculturalism) and use of CRPs (Morman et al., 2025). See Table 1 for overall sample means and measures included at each time point.

#### 2.2.2. Administrator Support for Educational Equity

The 2025 survey included two items used to measure district administrator support for equity and two items used to measure school administrator support originally used in the 2023 survey (Morman et al., 2025). Alongside these items, the 2025 survey included an additional five items assessing district-level administrator support and five items assessing school-level administrator support. Results of a confirmatory factor analysis supported a 2-factor model, in which school support (7 items) and district support (7 items) constituted separate factors and thus can be considered distinct constructs for the purpose of analyses: χ^2^(76) = 527.58, *p* < 0.001, CFI = 0.967; TLI = 0.961; RMSEA = 0.078; SRMR = 0.026.

#### 2.2.3. Voting-Based Community Political Climate

The 2025 survey operationalized community political climate in a comparable way to the 2023 survey, using community-level election data from the 2024 (versus 2020) presidential election. However, unlike the 2023 survey^2^, the 2025 survey did not include state-level DEI legislation when operationalizing community political climate, as all participants could be considered as living in a context that was not permissive of educational DEI efforts, given the existence of EO 14151 and subsequent actions and communication from the U.S. Department of Education, which applied across all states. Because this change in operationalization resulted in analyses that leveraged one community-level indicator of political climate (versus two indicators), we also adjusted the thresholds for community support, in alignment with prior research on community political climate (Woo et al., 2023). We considered contexts where the majority of individuals (>60%) voted for the 2024 Democratic presidential candidate to be liberal, and contexts where the majority (>60%) voted for the 2024 Republican presidential candidate to be conservative. Thus, in the 2025 survey, community-level political leanings served as a proxy for what we considered to be “DEI-permissive” vs. “DEI-opposed” in prior analyses of the 2023 survey. Polling data supports this operationalization, as it indicates that the majority of republicans oppose DEI efforts while the majority of democrats support these efforts (Bowman, 2025; Gecker & Sanders, 2025; Hu et al., 2025; Myeong, 2024).

### 2.3. Analytic Strategy

Analyses were designed to replicate and extend past research (Morman et al., 2025), exploring how the relationships between teachers’ individual moral frameworks (i.e., endorsement of multiculturalism and colorblindness), contextually derived frameworks (i.e., administrator support for equity and community political climate), and CRP use in 2025 compared to previous findings from 2023. First, replicating analyses of the 2023 survey, we conducted a latent profile analysis (LPA) using 2025 survey data to identify groups of teachers with differing moral frameworks regarding diversity (i.e., endorsement of multiculturalism and colorblindness). We compared the resulting profiles with those that emerged in analyses of the 2023 survey data. Then, we subset the 2025 data by LPA group and political climate to examine how the individual and contextual influences on teachers’ use of CRPs in 2025 compared to previous findings from 2023.

Finally, we conducted the same series of regression analyses within each data subset predicting CRP use from teachers’ endorsement of multiculturalism relative to colorblindness, administrator support for educational equity work, local political climate (i.e., DEI-opposed/conservative vs. DEI-permissive/liberal), and time point (i.e., 2023 vs. 2025). Regressions included covariates for teacher race and gender. We probed marginally and statistically significant interactions using simple slope analysis. All analyses were conducted in R 4.4.1 (R Core Team, 2025).

## 3. Results

### 3.1. LPA Replication: Profiles of Teachers’ Moral Frameworks Remained Stable in 2023 and 2025

**Figure 2:**
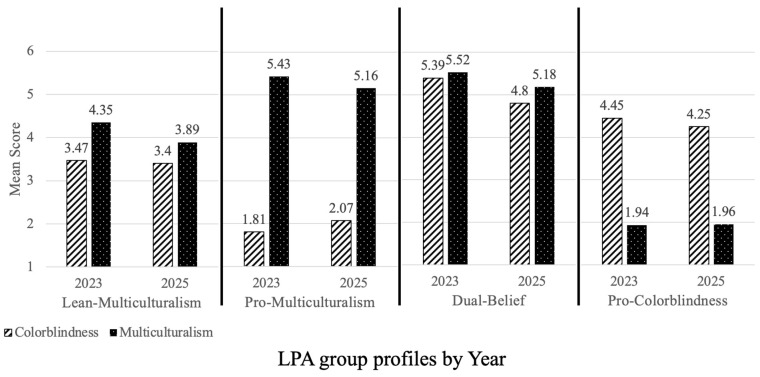
Mean multiculturalism and colorblindness scores for the four teacher groups identified through LPA using 2023 (as seen in Morman et al., 2025) and 2025 survey data.

The LPA using composite multiculturalism and colorblindness scores from the 2025 survey yielded the same four key profiles regarding teachers’ moral frameworks for diversity as emerged in analyses of the 2023 survey data (see Figure 2), with all indices suggesting that the model provided a good fit for the data (AIC = 5398.89, BIC = 5466.91; Entropy = 0.71; see Supplemental Materials Table S1 for additional profiles returned).

As in analyses of the 2023 survey, the lean-multiculturalism group (*N* = 385), included teachers who endorsed multiculturalism at moderately high levels (*M*_MC2025_ = 3.89; *SD*_MC2025_ = 0.53) but lacked a clear stance on colorblindness (*M*_CB2025_ = 3.4; *SD*_CB2025_ = 0.66). Repeated measures analyses indicated that these teachers’ mean scores for multiculturalism were significantly higher than their mean scores for colorblindness (*p* < 0.001). Although the pattern of endorsement replicated across samples, an ANOVA using a Bonferroni adjustment indicated that lean-multiculturalism teachers in 2025 endorsed multiculturalism (*F*(7, 1987) = 12.5, *p* < 0.001) slightly less than their counterparts in 2023, but were similar in their endorsement of colorblindness (*F*(7, 1987) = 1.52, *ns*). Lean-multiculturalism teachers comprised 39.3% of the 2025 sample compared to 62.7% of the 2023 sample.

The pro-multiculturalism group (*N* = 453) included teachers who strongly endorsed multiculturalism (*M*_MC2025_ = 5.16; *SD*_MC2025_ = 0.53) and weakly endorsed colorblindness (*M*_CB2025_ = 2.07; *SD*_CB2025_ = 0.65). Repeated measures analyses indicated that these teachers’ mean scores for colorblindness were significantly lower than their scores for multiculturalism (*p* < 0.001). While the pattern of endorsement replicated across samples, an ANOVA using a Bonferroni adjustment indicated that pro-multiculturalism teachers in 2025 endorsed multiculturalism slightly less (*F*(7, 1987) = 6.03, *p* < 0.001) and endorsed colorblindness slightly more (*F*(7, 1987) = −5.09, *p* < 0.001) than those in 2023. Pro-multiculturalism teachers comprised 46.2% of the 2025 sample compared to 26.4% of the 2023 sample.

The two smaller groups identified through the LPA comprised a similar percentage of the sample across studies (14.5% in 2025 and 10.8% in 2023). The dual-belief group (*N*_2023_ = 73 [7.1%], *N*_2025_ = 85 [8.4%]) included teachers who strongly endorsed both multiculturalism and colorblindness (*M*_MC2023_ = 5.52; *SD*_MC2023_ = 0.42; *M*_CB2023_ = 5.39; *SD*_CB2023_ = 0.40; *M*_MC2025_ = 5.18; *SD*_MC2025_ = 0.58; *M*_CB2025_ = 4.80; *SD*_CB2025_ = 0.63), although the mean score for multiculturalism was significantly higher than the mean score for colorblindness in both samples (*p* = 0.001). The pro-colorblindness group (*N*_2023_ = 37 [3.6%], *N*_2025_ = 57 [5.8%]) demonstrated low endorsement of multiculturalism and high endorsement of colorblindness (*M*_MC2023_ = 1.94; *SD*_MC2023_ = 0.58; *M*_CB2023_ = 4.45; *SD*_CB2023_ = 0.92; *M*_MC2025_ = 1.96; *SD*_MC2025_ = 0.56; *M*_CB2025_ = 4.25; *SD*_CB2025_ = 0.96; *p* < 0.001). Again, the pattern of endorsement replicated across samples, and an ANOVA using a Bonferroni adjustment indicated that dual-belief teachers in 2025 endorsed multiculturalism (*F*(7, 1987) = 3.64, *p* < 0.001) slightly less and colorblindness slightly more (*F*(7, 1987) = 5.65, *p* < 0.001) than their counterparts in 2023. The pro-colorblindness group in 2023 did not statistically differ from pro-colorblindness group in 2025 in their endorsement of either measure.

#### Demographic Variation Between Profiles

Table 2 displays the percentages of demographic variables within each group and overall for the 2023 and 2025 samples. The samples were comparable in terms of gender and race, but the 2025 sample included more teachers with a Master’s degree *X*^2^ (1, *N* = 1995) = 64.7, *p* < 0.001, and fewer teachers with an associate’s degree, *X*^2^ (1, *N* = 1995) = 95.2, *p* < 0.001, or doctoral degree, *X*^2^ (1, *N* = 1995) = 38.4, *p* < 0.001, compared to the 2023 sample. The 2025 lean-multiculturalism group included fewer Black/African American teachers, *X*^2^ (1, *N* = 1022) = 12.9, *p* < 0.001, and more White teachers, *X*^2^ (1, *N* = 1022) = 10.8, *p* < 0.001.

### 3.2. Changes in the Influence of Teachers’ Diversity Beliefs, Administrator Support, and Political Climate on Implementation of CRPs

To prepare the data for our focal analyses, we first subset the 2025 survey data by community political climate (i.e., liberal and conservative). We excluded teachers in locations with mixed political climates (i.e., communities where a presidential candidate received less than 60% of total votes, *N*_2025_ = 341), as it is unclear how these teachers perceived their local political climates in terms of support for DEI. As in the previous study, we excluded teachers in the dual-belief and pro-colorblindness groups due to insufficient sample sizes for statistical analyses. The analytic sample for the 2025 regression analysis included 315 teachers in liberal communities and 237 teachers in conservative communities. When combined with the 2023 sample, the final analytic sample included 586 teachers in liberal communities and 379 teachers in conservative communities. Although the analytic sample is notably smaller than the overall sample, its demographic composition and descriptive statistics of the focal variables were comparable to the full dataset (see Supplemental Materials Table S2).

We then combined the 2023 and 2025 datasets to examine whether the influence of individual and contextual frameworks on teachers’ use of CRPs differed as a function of time. Each community political climate by LPA group, resulting in four data subsets: teachers who weakly endorsed multiculturalism and taught in conservative communities, teachers who weakly endorsed multiculturalism and taught in liberal communities, teachers who strongly endorsed multiculturalism and taught in conservative communities, and teachers who strongly endorsed multiculturalism and taught in liberal communities. The decision to subset the data in this way aligns with longitudinal approaches that aim to observe change over time by comparing groups of people who have similar contexts and experiences at different points in time (Bieler et al., 2010).

We conducted a series of stepwise regression analyses to investigate whether the influence of teachers’ diversity beliefs, administrators’ support for equity, and political climate on CRP use differed in 2025 compared to 2023, using z-scores for school and district support variables^3^ to account for differences in the number of items included within the composite. We ran separate regression analyses for each group to explore whether the influence of district administrator support on teachers’ use of CRPs changed before and after the federal-level anti-DEI EO comprise Models 1–4, included within Table 3. Regression analyses for the influence of school administrator support differed before and after the federal-level anti-DEI EO comprise Models 5–8, included within Table 4. Within each table, the models are presented first by teacher diversity belief group and then by community political climate.^4^ Effects for lean-multiculturalism teachers in conservative communities are displayed in Models 1 and 5, lean-multiculturalism teachers in liberal communities in Models 2 and 6, pro-multiculturalism teachers in conservative communities in Models 3 and 7, and pro-multiculturalism teachers in liberal communities Models 4 and 8. For simplicity, we report only interactions within the main text (see Supplemental Materials Table S3 for main effects by group).

**Figure 3:**
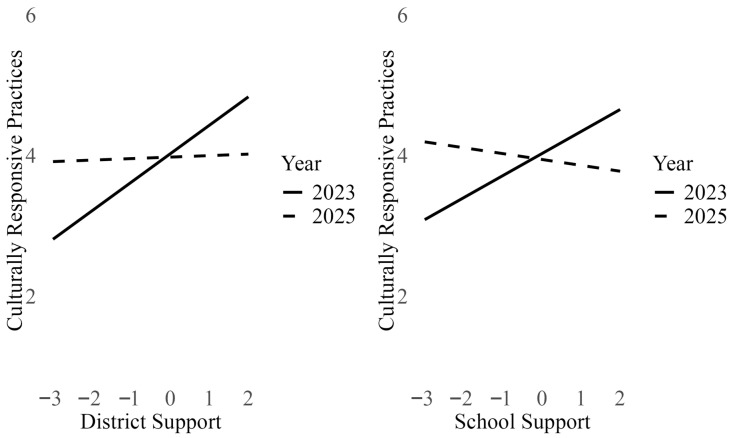
Influence of administrator support for equity work on culturally responsive practice use among lean-multiculturalism teachers in conservative communities (Models 1 and 5, respectively).

Only lean-multiculturalism teachers in conservative communities demonstrated a different pattern in 2025 compared to 2023 in terms of how their individual and contextual frameworks influenced their use of CRPs (Models 1 and 5). As shown in Figure 3, lean-multiculturalism teachers in conservative communities in 2023 (solid line) reported using CRPs more frequently when they perceived greater district (*B* = 0.41, *p* < 0.01) or school (*B* = 0.31, *p* = 0.01) administrator support for equity work. However, for lean-multiculturalism teachers in similar communities in 2025 (dashed line), district (*B* = 0.02, *p* = 0.83) and school administrator (*B* = −0.08, *p* = 0.44) support for equity work did not predict CRP use. There were no differences in the effects of school and district administrator support on CRP use among lean-multiculturalism teachers in liberal communities in 2023 vs. 2025 (Models 2 and 6). Across both years, school (*Bs* = 0.29–0.38, *ps* < 0.05–0.001) and district (*Bs* = 0.34–0.39, *ps* < 0.05–0.001) administrator support predicted greater CRP use.

For pro-multiculturalism teachers, there was no difference between 2023 and 2025 in terms of the influence of individual and contextual moral frameworks on CRP use, regardless of community political climate (Models 3, 4, 7, and 8). In liberal communities, neither district (Model 4) nor school administrator (Model 8) support for equity predicted greater likelihood of CRP use among these teachers (*B*s = 0.01–0.02, *ns*), while in conservative communities, both district (Model 3) and school (Model 7) support for equity work predicted greater CRP use (*B*s = 0.60–0.72, *p*s < 0.01–0.001).

## 4. Discussion

Results of this study point to both stability and changes in the ways teachers’ individual and contextually derived moral frameworks influence their decisions to use CRPs in the wake of changing federal guidance regarding educational DEI efforts. First, across both time points (2023 and early 2025), the same four profiles emerged to characterize the way teachers make sense of diversity efforts in educational contexts. The most common profile included teachers who endorsed multiculturalism at moderately high levels but lacked a clear stance on colorblindness (i.e., lean-multiculturalism teachers) and thus held relatively less codified or more mixed moral frameworks regarding the role of diversity in education. The second most common profile included teachers who strongly endorsed multiculturalism and weakly endorsed colorblindness (i.e., pro-multiculturalism teachers) and thus held relatively more codified moral frameworks that suggest it is important to attend to issues of diversity in educational contexts. These two profiles comprised the majority (87%) of both the 2023 and 2025 samples and thus were the focal groups for analyses. While the pattern of endorsement of multiculturalism and colorblindness remained stable across both times, teachers’ endorsement of the key diversity ideologies differed across time. Pro-multiculturalism teachers reported lower endorsement of both multiculturalism and colorblindness in 2025 compared to 2023. Lean-multiculturalism teachers report lower endorsement of multiculturalism and higher endorsement of colorblindness more in 2025 compared to 2023. While further research is needed to understand the generalizability and cause of these changes, it is possible that changes in political landscape have led teachers, particularly those who more strongly believe in the importance of acknowledging diversity in educational contexts, to temper their expression of their beliefs (see Borsheim-Black, 2024). At the same time, the greater endorsement of colorblindness among lean-multiculturalism teachers could reflect a greater willingness or propensity to adopt a stance toward diversity that aligns with new federal guidance indicating that schools should not prioritize these issues.

Second, results largely conveyed stability in the way individual and contextual moral frameworks shape teachers’ decisions to use CRPs, particularly among teachers with more codified individual moral frameworks about diversity (i.e., pro-multiculturalism teachers). In both 2023 and 2025, pro-multiculturalism teachers in liberal communities, where the contextual frameworks regarding diversity generally aligned with their individual frameworks, reported relatively frequent use of CRPs, regardless of their school or district administrators’ support for equity work. In contrast, when pro-multiculturalism teachers worked in conservative communities, where the contextual frameworks regarding diversity differed from their own, they relied on guidance from school or district administrators to inform their use of CRPs in 2023 and 2025. In other words, at both time points, pro-multiculturalism teachers implemented practices that aligned with their individual moral frameworks when they taught in communities where people generally shared their perspectives about diversity. When they taught in communities that held differing beliefs about diversity, however, pro-multiculturalism teachers relied more heavily on guidance from their school and district administrators, implementing CRPs more often when administrators were supportive of these practices and less often when administrators were not supportive.

The key difference between results from studies conducted in 2023 and 2025 concerned the factors influencing CRP use among teachers with less-codified individual frameworks regarding diversity (i.e., lean-multiculturalism teachers). Prior to changes in federal guidance regarding educational DEI efforts, lean-multiculturalism teachers demonstrated a similar pattern regardless of the type of community they worked in: as administrators’ support for equity work increased, so did their use of CRPs. Following changes in federal guidance, however, administrator support was only influential among lean-multiculturalism teachers who taught in liberal communities. Because the results from 2023 and 2025 were derived from different teachers, we cannot draw causal conclusions about the effects of changing federal guidance on individual teachers’ decisions regarding CRP implementation. However, the group-level patterns indicate that, when determining which practices to use with their students, teachers with weaker multiculturalism beliefs in 2025 (i.e., those who may be more inclined to agree with new federal guidance to deprioritize or suspend DEI efforts) who taught in conservative communities were less influenced by school or district leaders’ support for equity work compared to their counterparts in 2023.

### Limitations and Future Directions

By leveraging large national samples of different K-12 educators, this study provides further support for the notion that educators weigh a combination of individual and contextual factors when deciding which practices to use to engage their students. Both prior to and following changing federal guidance regarding DEI, educators self-reported responses indicated that they consistently looked to their administrators and communities to inform their practices, particularly when their individual beliefs about diversity differed from the prevailing beliefs within their communities. These findings, however, should be considered alongside the methodological limitations of this study. First, while we identified ways in which the individual and contextual influences on teachers’ practice decisions remained consistent or changed following changing federal guidance, the two samples reported in this paper include different individuals. Thus, at an individual level, it is unclear whether and how federal guidance has affected teachers’ decision making. It is also unclear to what extent our reliance on self-reported measures of key predictor variables (i.e., district and school support) and outcome variables (i.e., use of CRPs) may have introduced common method bias. Instead, the results should be interpreted through the diversity beliefs profiles, which replicated across both time points and suggest that teachers who hold certain constellations of individual beliefs about diversity are likely to respond similarly to the same contextual factors across time.

Second, this study relied on teacher-reported school and district support for equity work and may not fully capture how administrators enact policy into everyday norms and processes. Examining other measures, such as administrators’ own reports of their beliefs, policies, or practices related to equity could better elucidate the relationship between administrator support for equity work and teachers’ decisions regarding CRPS, as well as the types of administrator support that prove particularly influential. Moreover, it is possible that other contextual influences within schools, beyond perceived administrator support, influence teachers’ use of CRPs. For example, prior research illustrates that the structures surrounding teaching and learning, such as curricula (Leithwood, 2021) and professional development (Brady et al., 2025), can influence teachers’ decisions about which instructional practices they use to engage students. Because these structures are often intertwined with and bolstered by district and school administrators’ support for equity (Brady et al., 2024; Peurach et al., 2019; Stevens et al., 2025), it may be fruitful for future research to explore the interplay between administrators’ social and structural support for equity on teachers’ CRP use.

Lastly, this paper operationalized political climate using proxy measures that relied partially or entirely upon community-level voting behavior (i.e., a combination of state-level anti-DEI laws and voting in 2023, and voting in 2025). While these proxies offer objective measures of political behavior and consistent measurement across time (see Supplemental Materials Tables S4 and S5), they do not fully capture the complex nature of a community’s political climate. Future research should consider leveraging a wider range of measures of political climate, for example, by integrating measures of school, district, or municipal policy.

## 5. Conclusions

The public conversation about educational DEI efforts has become increasingly contentious and confusing in recent years. For many teachers, recent changes at the federal level regarding the role of DEI in education and the potential consequences for those who support DEI efforts have created an added layer of concern. The current study provides timely insights into how educators are navigating this particular political moment. Despite concerns among some opponents of educational DEI initiatives, the findings do not suggest that teachers are primarily ideologically motivated when determining how to engage students. Instead, they indicate that teachers are consistently attending to and using information about their communities’ preferences to inform their practices. Moreover, this is particularly true when educators’ own beliefs differ from those of their communities, regardless of how inclined or disinclined teachers are to view diversity as an important consideration in educational contexts. As educators navigate a changing landscape, the results also suggest that administrators can play a key role in helping educators navigate practice-related decisions. In this uncertain climate, the role of administrators as advocates for equity may prove decisive in determining whether CRPs persist or diminish within teachers’ classroom practices. Whether administrators can reframe the challenge of a changing landscape as manageable—not insurmountable—remains to be seen.

## Figures and Tables

**Table 1:** Key measures, sample items, and means for 2023 and 2025 surveys.

Measure	Example Item and Scale	2023 M (SD)	2025 M (SD)	Overall
Culturally responsive practices (5 items, α_2023_ = 0.84; α_2025_ = 0.81)	“Use the cultural background of my students to make learning meaningful” (1 [Never] to 6 [A few times a day])	4.05 _a_ (1.20)	4.22 _b_ (1.22)	4.13 (1.21)
Multiculturalism diversity beliefs (5 items, α_2023_ = 0.86, α_2025_ = 0.85)	“Teachers should use instructional materials that reflect the racial and ethnic diversity of their students” (1 [Strongly Disagree] to 6 [Strongly Agree])	4.63 _a_ (0.96)	4.47 _b_ (1.02)	4.55 (0.99)
Colorblindness diversity beliefs (4 items, α_2023_ = 0.76, α_2025_ = 0.78)	“Given the diversity of students, educators should encourage racial and ethnic minorities to adapt to mainstream expectations of society” (1 [Strongly Disagree] to 6 [Strongly Agree])	3.20 _a_ (1.17)	2.96 _b_ (1.13)	3.08 (1.16)
District administrator support for educational equity (2023: 2 items, α = 0.82; 2025: 7 items, α = 0.96)	“My district prioritizes equity work” (1 [Strongly Disagree] to 6 [Strongly Agree])	4.23 _a_ (1.22)	4.20 _a_ (1.11)	4.22 (1.16)
School administrator support for educational equity (2023: 2 items, α = 0.79; 2025: 7 items, α = 0.95)	“My school prioritizes equity work” (1 [Strongly Disagree] to 6 [Strongly Agree])	4.55 _a_ (1.12)	4.32 _b_ (1.12)	4.43 (1.13)
		%	%	
Voting-based political climate: Permissive vs. Opposed	Permissive: >50% votes for 2020 Democratic candidate + no state-level anti-DEI legislation Opposed: >50% votes for 2020 Republican candidate + anti-DEI legislation	34.2%		
Voting-base political climate: Liberal vs. Conservative	Liberal: >60% votes for 2024 Democratic candidate Conservative: >60% votes for 2024 Republican Candidate		43.2%	

Note: Differing subscripts for 2023 and 2025 means indicate statistically significant differences at *p* < 0.05.

**Table 2:** Demographic characteristics of the four identified teacher groups and the overall sample by each time point.

Demographic Characteristic		Lean-Multiculturalism		Pro-Multiculturalism		Dual-Belief		Pro-Colorblindness		Overall	
		2023 (*N* = 637)	2025 (*N* = 385)	2023 (*N* = 268)	2025 (*N* = 453)	2023 (*N* = 73)	2025 (*N* = 85)	2023 (*N* = 37)	2025 (*N* = 57)	2023 (*N* = 1015) ^1^	2025 (*N* = 980)
Gender ^2^	Male	23.1% _a_	21.0% _a_	12.3% _a_	17.0% _a_	17.8% _a_	22.4% _a_	37.8% _a_	33.3% _a_	20.4% _a_	20.0% _a_
	Female	76.8% _a_	77.1% _a_	86.6% _a_	81.2% _a_	83.6% _a_	76.5% _a_	62.2% _a_	66.7% _a_	79.3% _a_	78.4% _a_
Race ^3^	White	77.8% _a_	86.2% _b_	79.1% _a_	75.7% _a_	56.2% _a_	55.3% _a_	94.6% _a_	86.0% _a_	77.1% _a_	78.7% _a_
	Black/African American	12.6% _a_	5.2% _b_	13.4% _a_	13.5% _a_	17.8% _a_	30.6% _a_	0% _a_	0% _a_	12.7% _a_	10.9% _a_
	Hispanic/Latine American	9.9% _a_	7.3% _a_	7.8% _a_	9.3% _a_	13.7% _a_	16.5% _a_	2.7% _a_	8.8% _a_	9.4% _a_	9.1% _a_
	Asian American	4.7% _a_	5.2% _a_	3.4% _a_	5.3% _a_	8.2% _a_	7.1% _a_	2.7% _a_	1.8% _a_	4.5% _a_	5.2% _a_
	Native American	1.7% _a_	1.6% _a_	1.9% _a_	2.0% _a_	2.7% _a_	3.5% _a_	8.1% _a_	5.3% _a_	2.1% _a_	2.1% _a_
Education	2-year degree (associate’s)	16.0% _a_	2.3% _b_	7.5% _a_	0.9% _b_	17.8% _a_	4.7% _b_	5.4% _a_	0% _a_	13.5% _a_	1.7% _b_
	4-year degree (bachelor’s)	42.4% _a_	40.5% _a_	35.1% _a_	35.1% _a_	48.0% _a_	49.4% _a_	46.0% _a_	38.6% _a_	41.0% _a_	38.7% _a_
	Master’s degree	38.0% _a_	57.1% _b_	54.9% _a_	64.0% _b_	24.7% _a_	45.9% _b_	37.8% _a_	61.4% _b_	41.5% _a_	59.6% _b_
	Doctoral degree	3.6% _a_	0% _b_	2.6% _a_	0% _b_	9.6% _a_	0% _b_	10.8% _a_	0% _b_	4% _a_	0% _b_

^1^ Within the 2025 sample, inclusion criteria for education were limited to those with an associate’s degree or higher. This inclusion was retroactively applied to the 2023 sample. ^2^ Nine teachers identified as nonbinary: five were in Group 1 and four were in Group 2. Three teachers identified as transgender: one was in Group 1 and two were in Group 2. ^3^ Four teachers identified as Middle Eastern/North African: two were in Group 1, one in Group 2, and one in Group 4. Note: Differing subscripts for 2023 and 2025 means indicate statistically significant differences at *p* < 0.05.

**Table 3:** Regression analyses examining the influence of individual (i.e., diversity beliefs) and contextual (i.e., district administrator support) frameworks on teachers’ use of culturally responsive practices before and after changes in federal guidance regarding educational diversity, equity, and inclusion efforts. Model 1: lean-multiculturalism teachers in conservative communities (*N* = 252); model 2: lean-multiculturalism teachers in liberal communities (*N* = 299); model 3: pro-multiculturalism teachers in conservative communities (*N* = 127); model 4: pro-multiculturalism teachers in liberal communities (*N* = 287).

	Interaction of New Federal Guidance & District Administrator Support			
	Lean-Multiculturalism Teachers		Pro-Multiculturalism Teachers	
	Conservative Communities (*N* = 252)	Liberal Communities (*N* = 299)	Conservative Communities (*N* = 127)	Liberal Communities (*N* = 287)
	Model 1	Model 2	Model 3	Model 4
Intercept	3.65 *** (0.17)	4.05 *** (0.14)	4.66 *** (0.30)	4.10 *** (0.18)
Race (0 = White; 1 = non-White)	0.11 (0.22)	−0.07 (0.15)	0.03 (0.25)	0.28 * (0.14)
Gender (0 = Male; 1 = non-Male)	0.35 (0.18)	0.10 (0.15)	−0.36 (0.27)	0.01 (0.17)
New Federal Guidance (0 = 2023; 1 = 2025)	0.04 (0.15)	−0.04 (0.13)	0.03 (0.21)	0.18 (0.14)
District Support	0.39 ** (0.12)	0.34 *** (0.08)	0.72 *** (0.18)	0.01 (0.10)
New Federal Guidance * District Support	−0.37 * (0.15)	−0.15 (0.13)	−0.33 (0.21)	0.18 (0.13)
R^2^	0.07	0.07	0.22	0.04

*** *p* < 0.001; ** *p* < 0.01; * *p* < 0.05.

**Table 4:** Regression analyses examining the influence of individual (i.e., diversity beliefs) and contextual (i.e., school administrator support) frameworks on teachers’ use of culturally responsive practices before and after changes in federal guidance regarding educational diversity, equity, and inclusion efforts. Model 5: lean-multiculturalism teachers in conservative communities (*N* = 252); model 6: lean-multiculturalism teachers in liberal communities (*N* = 299); model 7: pro-multiculturalism teachers in conservative communities (*N* = 127); model 8: pro-multiculturalism teachers in liberal communities (*N* = 287).

	Interaction of New Federal Guidance & School Administrator Support			
	Lean-Multiculturalism Teachers		Pro-Multiculturalism Teachers	
	Conservative Communities (*N* = 252)	Liberal Communities (*N* = 299)	Conservative Communities (*N* = 127)	Liberal Communities (*N* = 287)
	Model 5	Model 6	Model 7	Model 8
Intercept	3.65 *** (0.18)	4.05 *** (0.14)	4.71 *** (0.32)	4.11 *** (0.18)
Race (0 = White; 1 = non-White)	0.13 (0.22)	−0.07 (0.15)	0.03 (0.26)	0.26 (0.14)
Gender (0 = Male; 1 = non-Male)	0.38 * (0.18)	0.09 (0.15)	−0.47 (0.28)	0.02 (0.17)
New Federal Guidance (0 = 2023; 1 = 2025)	−0.00 (0.15)	−0.02 (0.13)	0.08 (0.22)	0.19 (0.14)
School Support	0.29 * (0.11)	0.38 *** (0.08)	0.60 ** (0.19)	0.03 (0.17)
New Federal Guidance * School Support	−0.37 * (0.15)	−0.20 (0.13)	−0.33 (0.22)	0.04 (0.13)
R^2^	0.06	0.08	0.15	0.02

*** *p* < 0.001; ** *p* < 0.01; * *p* < 0.05.

## Data Availability

The data presented in this study are available on request from the corresponding author due to their use in ongoing research.

## Notes

1 Within the 2025 sample, inclusion criteria for education was limited to those with an associate’s degree. This inclusion was retroactively applied to the 2023 sample.

2 Analyses of the 2023 survey used a combination of state-level anti-DEI legislation and community-level 2020 election data to create an indicator of community political climate. Communities without state-level legislation restricting DEI efforts and where more than 50% of voters supported the 2020 Democratic presidential candidate were considered “DEI-permissive.” Communities with state-level legislation restricting DEI efforts and where less than 50% of voters supported the 2020 Democratic presidential candidate were considered “DEI-opposed.” Exploratory comparison of these two proxy measures indicated a 1-to-1 match between those included in the analytic sample of (Morman et al., 2025).

3 Due to strong correlation between the two types of administrator support (r = 0.74, *p* < 0.001), we estimated separate regression models for district support and school support to avoid suppression effects (Pandey & Elliott, 2010; Smith et al., 1992).

4 To increase readability, we use the term “conservative communities” to refer to DEI-opposed communities in the 2023 dataset and politically conservative communities in the 2025 dataset.

## Supplementary Materials

The following supporting information can be downloaded at: https://www.mdpi.com/article/10.3390/bs16030390/s1. Reference Akogul and Erisoglu (2017) is cited in the supplementary materials.
