# Supplementary material for: Change in the Air: How Shifting Federal Guidance Related to DEI Influences Teachers’ Use of Culturally Responsive Practices
Source: Behav Sci (Basel). 2026 Mar 9;16(3):390. doi: 10.3390/bs16030390 (PMC13024554; doi:10.3390/bs16030390)
Supplement: Supplementary file 1 [file behavsci-16-00390-s001.zip › behavsci-4081122-supplementary.pdf]

Supplemental Materials

Table S1. Model fit indices for differing classes returned for latent profile classes.

Model	Classes	AIC	BIC	Entropy
**EEE**	2	5515.83	5554.94	0.83
	3	5427.54	5481.31	0.83
	4	5398.48	5466.91	0.71
	5	5403.37	5486.46	0.58
	6	5388.89	5486.64	0.63

Note: An analytic hierarchy process, based on the fit indices of AIC, BIC and Entropy (Akogul & Erisoglu, 2017), suggests the best solution is model 3 with 4 classes. Lower scores for AIC and BIC indicate more parsimony between complexity and fit, while higher entropy indicates better distinction between classes. When AIC and BIC differ in identifying the same model, entropy values and a threshold of 0.70 should be used to evaluate the distinctness of the model. Thus, the model with 4 classes, which has the second lowest AIC, the lowest BIC, and an entropy above 0.70 was selected. Notably, a degree of overlap in group classification exists within all LPA profiles. An entropy value of 0.71 is on the lower end of the acceptable range, which suggests that there was some amount of uncertainty in assigning participants to a specific class. Because LPA profiles are used as predictors in subsequent regression analyses, this uncertainty may introduce variance in the estimates resulting from the main analyses.

Table S2. Comparison of demographic characteristics and descriptive statistics between the overall sample and the analytical sample.

		**Overall Sample**				**Analytic Sample**			
		**Lean-multiculturalism**		**Pro-multiculturalism**		**Lean-multiculturalism**		**Pro-multiculturalism**	
		**2023 (*N* = 637)**	**2025 (*N* = 385)**	**2023 (*N* = 268)**	**2025 (*N* = 453)**	**2023 (*N* = 291)**	**2025 (*N* = 260)**	**2023 (*N* = 137)**	**2025 (*N* = 277)**
Gender	Male	23.1%	21.0%	12.3%	17.0%	25.1%	21.5%	13.9%	17.7%
	Female	76.8%	77.1%	86.6%	81.2%	74.6%	76.2%	84.7%	80.1%
Race	White	77.8%	86.2%	79.1%	75.7%	81.4%	86.5%	82.5%	71.5%
	Black/African American	12.6%	5.2%	13.4%	13.5%	13.8%	5.4%	13.9%	16.3%
	Hispanic/Latine American	9.9%	7.3%	7.8%	9.3%	6.9%	9.2%	3.7%	10.5%

	Asian	4.7%	5.2%	3.4%	5.3%	5.2%	5.0%	5.1%	5.1%
	Native American	1.7%	1.6%	1.9%	2.0%	2.8%	1.5%	1.5%	2.5%
Education	2-year college degree (associate's degree)	16.0%	2.3%	7.5%	0.9%	12.4%	1.5%	5.8%	1.1%
	4-year bachelor's degree	42.4%	40.5%	35.1%	35.1%	44.3%	41.9%	32.9%	36.8%
	Master's degree	38.0%	57.1%	54.9%	64.0%	40.2%	56.5%	57.7%	62.1%
	Doctoral degree	3.6%	0%	2.6%	0%	3.1%	0%	3.7%	0%
Focal variables	Multiculturalism	4.35 (0.70)	3.89 (0.52)	5.43 (0.44)	5.16 (0.53)	4.38 (0.71)	3.89 (0.52)	5.43 (0.45)	5.15 (0.53)
	Colorblindness	3.47 (0.71)	3.40 (0.65)	1.81 (0.47)	2.07 (0.65)	3.43 (0.70)	3.40 (0.66)	1.77 (0.47)	2.02 (0.64)
	Culturally Responsive Practices	3.98 (1.17)	4.03 (1.16)	4.24 (1.09)	4.43 (1.13)	4.06 (1.18)	4.03 (1.14)	4.21 (1.12)	4.41 (1.11)
	District Support	4.22 (1.10)	4.03 (0.99)	4.16 (1.3)	4.28 (1.15)	4.28 (1.04)	4.01 (1.03)	4.24 (1.24)	4.27 (1.13)
	School Support	4.53 (0.95)	4.13 (0.99)	4.55 (1.29)	4.47 (1.13)	4.57 (0.95)	4.13 (1.00)	4.58 (1.21)	4.42 (1.14)

Main Effect Influence of Federal Restrictions and Administrator Support Among Teachers with Similar Diversity Beliefs in Similar Community Political Climates

Table S3. Main effects regression analyses examining the influence of individual and contextual frameworks on teachers' use of culturally responsive practices before and after changes in federal guidance regarding educational diversity, equity, and inclusion efforts. Models 1-3: lean-multiculturalism teachers in conservative communities (*N* = 255); models 4-6: lean-multiculturalism teachers in liberal communities (*N* = 301); models 7-9: pro-multiculturalism teachers in conservative communities (*N* = 126); models 10-12: pro-multiculturalism teachers in liberal communities (*N* = 286).

Lean-multiculturalism teachers	Pro-multiculturalism teachers

	Conservative communities (*N* = 255)		Liberal communities (*N* = 301)		Conservative communities (*N* = 126)		Liberal communities (*N* = 286)	
	**Main Effects**		**Main Effects**		**Main Effects**		**Main Effects**	
	**Model 1**	**Model 2**	**Model 3**	**Model 4**	**Model 5**	**Model 6**	**Model 7**	**Model 8**
Intercept	3.68*** (0.18)	3.65*** (0.18)	4.01*** (0.14)	4.04*** (0.14)	4.09*** (0.18)	4.10*** (0.18)	4.70*** (0.30)	4.72*** (0.32)
Race (0 = White; 1 = non-White)	0.17 (0.22)	0.19 (0.22)	-0.05 (0.15)	-0.06 (0.15)	0.28 (0.14)	0.26 (0.14)	0.03 (0.25)	0.02 (0.26)
Gender (0 = Male; 1 = non-Male)	0.41 *(0.18)	0.43 *(0.18)	0.10 (0.15)	0.09 (0.15)	0.02 (0.17)	0.02 (0.17)	-0.38 (0.27)	-0.47 (0.28)
New Federal Guidance (0 = 2023; 1 = 2025)	0.02 (0.15)	-0.02 (0.15)	-0.04 (0.13)	-0.02 (0.13)	0.17 (0.14)	0.19 (0.14)	0.03 (0.20)	0.07 (0.22)
District Support	0.17 *(0.08)		0.33*** (0.07)		0.11 (0.06)		0.43*** (0.08)	
School Support		0.09 (0.08)		0.33*** (0.07)		0.05 (0.06)		0.33*** (0.09)
R^2	0.03	0.02	0.06	0.06	0.02	0.01	0.18	0.10

*** p < 0.001; ** p < 0.01; * p < 0.05

Replication of Regression Analysis with the 2025 Measure of Voting-Based Effect Influence of Federal Restrictions and Administrator Support Among Teachers with Similar Diversity Beliefs in Similar Community Political Climate Retroactively Applied to the 2023 Sample

Table S4. Regression analyses examining the influence of individual (i.e., diversity beliefs) and contextual (i.e., district administrator support) frameworks on teachers' use of culturally responsive practices before and after changes in federal guidance regarding educational diversity, equity, and inclusion efforts with the 2025 measure

of voting-based political climate retroactively applied to the 2023 sample.

Note: In recombining the 2023 sample with the 2025 sample, 67 teachers with a community political climate previously operationalized as “Mixed” could be reclassified as either “Liberal” (*N* = 28) or “Conservative” (*N* = 39). We chose to include these individuals within this measure replication.

Interaction of New Federal Guidance & District Administrator Support

	**Lean-multiculturalism teachers**		**Pro-multiculturalism teachers**	
	Conservative communities (*N* = 281)	Liberal communities (*N* = 324)	Conservative communities (*N* = 137)	Liberal communities (*N* = 290)
	Model 9	Model 10	Model 11	Model 12
Intercept	3.81 *** (0.16)	4.10 *** (0.13)	4.71 *** (0.29)	4.18 *** (0.14)
Race (0 = White; 1 = non-White)	0.06 (0.21)	0.05 (0.14)	−0.06 (0.26)	0.25 (0.14)
Gender (0 = Male; 1 = non-Male)	0.18 (0.17)	0.03 (0.14)	−0.41 (0.27)	0.03 (0.17)
New Federal Guidance (0 = 2023; 1 = 2025)	0.01 (0.14)	−0.06 (0.13)	0.03 (0.19)	0.10 (0.14)
District Support	0.35 *** (0.10)	0.28 *** (0.08)	0.73 *** (0.16)	0.07 (0.10)
New Federal Guidance * District Support	−0.33 * (0.14)	−0.08 (0.13)	−0.33 (0.19)	0.11 (0.13)
R^2	0.05	0.05	0.23	0.03
*** p < 0.001; ** p < 0.01; * p < 0.05				

Table S5. Regression analyses examining the influence of individual (i.e., diversity beliefs) and contextual (i.e., school administrator support) frameworks on teachers’ use of culturally responsive practices before and after changes in federal guidance regarding educational diversity, equity, and inclusion efforts with the 2025 measure of voting-based political climate retroactively applied to the 2023 sample.

Note: In recombining the 2023 sample with the 2025 sample, 67 teachers with a community political climate previously operationalized as “Mixed” could be reclassified as either “Liberal” (*N* = 28) or “Conservative” (*N* = 39). We chose to include these individuals within this measure replication.

	Interaction of New Federal Guidance & District Administrator Support			
	**Lean-multiculturalism teachers**		**Pro-multiculturalism teachers**	
	Conservative communities (*N* = 281)	Liberal communities (*N* = 324)	Conservative communities (*N* = 137)	Liberal communities (*N* = 290)
	Model 13	Model 14	Model 15	Model 16
Intercept	3.83 *** (0.16)	4.09 *** (0.13)	4.78 *** (0.30)	4.19 *** (0.18)
Race (0 = White; 1 = non-White)	0.03 (0.21)	0.06 (0.14)	0.02 (0.26)	0.23 (0.14)
Gender (0 = Male; 1 = non-Male)	0.17 (0.17)	0.02 (0.14)	−0.48 (0.28)	0.03 (0.17)
New Federal Guidance (0 = 2023; 1 = 2025)	−0.01 (0.14)	−0.04 (0.13)	0.01 (0.20)	0.11 (0.14)
District Support	0.36 *** (0.10)	0.28 *** (0.08)	0.66 *** (0.15)	0.09 (0.10)
New Federal Guidance * District Support	−0.44 ** (0.14)	−0.10 (0.13)	−0.37 (0.19)	−0.02 (0.13)
R^2	0.05	0.05	0.19	0.02

*** $p < 0.001$; ** $p < 0.01$; * $p < 0.05$
